# Rehabilitation of amblyopia using a digital platform for visual training combined with patching in children: a prospective study

**DOI:** 10.1007/s00417-024-06475-0

**Published:** 2024-04-05

**Authors:** Carlos J. Hernández-Rodríguez, Patricia Ferrer-Soldevila, Alberto Artola-Roig, David P. Piñero

**Affiliations:** 1 Group of Optics and Visual Perception, Department of Optics, Pharmacology and Anatomy, University of Alicante, Crta San Vicente del Raspeig S/N 03016, San Vicente del Raspeig, Alicante, Spain; 2 Clinical Optometry Unit, Department of Ophthalmology, Ribera Virgen de La Caridad Hospital, Cartagena, Spain; 3 Department of Ophthalmology, Ribera Virgen de La Caridad Hospital, Cartagena, Spain; 4 Department of Ophthalmology, Vithas Medimar International Hospital, Alicante, Spain; 5 Clinical Optometry Unit, Department of Ophthalmology, Vithas Medimar International Hospital, Alicante, Spain

**Keywords:** Amblyopia, Perceptual learning, Dichoptic therapy, Patching, Visual training

## Abstract

**Purpose:**

To assess the possible benefits of the use of perceptual learning and dichoptic therapy combined with patching in children with amblyopia over the use of only patching.

**Methods:**

Quasi-experimental multicentric study including 52 amblyopic children. Patients who improved their visual acuity (VA) by combining spectacles and patching were included in patching group (PG: 20 subjects), whereas those that did not improved with patching performed visual training (perceptual learning + dichoptic therapy) combined with patching, being assigned to the visual treatment group (VT: 32 subjects). Changes in VA, contrast sensitivity (CS), and stereopsis were monitored during a 6-month follow-up in each group.

**Results:**

Significant improvements in VA were found in both groups at 1 month (*p* < 0.01). The total improvement of VA was 0.18 ± 0.16 and 0.31 ± 0.35 logMAR in PG and VT groups, respectively (*p* = 0.317). The Wilcoxon effect size was slightly higher in VT (0.48 vs. 0.54) at 6 months. An enhancement in CS was observed in the amblyopic eye of the VT group for all spatial frequencies at 1 month (*p* < 0.001). Likewise, the binocular function score also increased significantly in VT group (*p* = 0.002). A prediction equation of VA improvement at 1 month in VT group was obtained by multiple linear regression analysis (*p* < 0.001, *R*^2^ = 0.747).

**Conclusions:**

A combined treatment of visual training and patching is effective for obtaining a predictable improvement of VA, CS, and binocularity in patching-resistant amblyopic children.

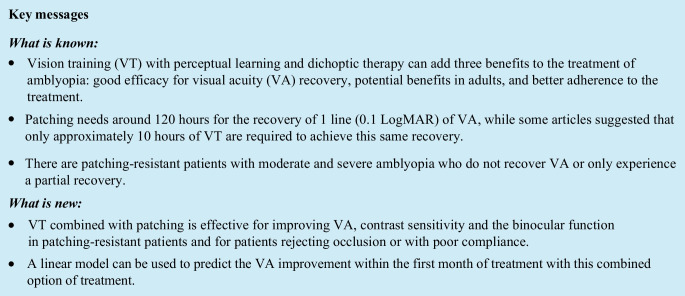



## Introduction

Amblyopia is a visual neurodevelopmental disorder due to a disruption of binocular vision during early years, with a prevalence of around 3.4% of the pediatric population [1]. The main characteristic of amblyopic subjects is a decreased best-corrected visual acuity (VA) and contrast sensitivity (CS) in one eye, or rarely in both, combined with reduced or null stereopsis [2, 3]. Also, amblyopic eyes show accommodative [4, 5], fixation and oculomotor disorders [6, 7], as well as many others deficits in visual processing and perception [8]. In addition, some of these signs can be also found in the fellow eye [9]. According to its etiology, amblyopia can be classified into three categories: refractive amblyopia, which includes anisometropic amblyopia (differences between eyes of > 1.0 D in hyperopia or > 5.0 D in myopia in sphere and > 1.5 D in cylinder) and isometropic amblyopia (differences between eyes less than 1.0 D in sphere and 1.5 D in cylinder), strabismic amblyopia (if there is a tropia or microtropia), or mixed amblyopia (if anisometropia and strabismus coexist) [10]. Another infrequent type of amblyopia is deprivation amblyopia, which is caused by losses of transparency of ocular media or any other obstacle avoiding light reaching retina, being commonly associated to a severe visual deficit [10]. Also, amblyopia classification can be performed according to severity in mild amblyopia if visual acuity ranges from 0.1 logMAR to 0.2 logMAR, moderate amblyopia from 0.3 logMAR to 0.6 logMAR, and severe amblyopia from 0.7 logMAR to 1.30 logMAR in the worse eye.

The main treatment for amblyopia is the compensation of the refractive error in combination with occlusion of the fellow eye when needed. Notwithstanding glasses and patching are the gold standard with demonstrated efficacy [11–13], but there are some critical aspects to consider about occlusion. First, there are patching-resistant patients with moderate and severe amblyopia who do not recover VA or only experience a partial recovery [11]. Second, patching is a passive treatment with no direct stimulation of binocularity, with a little effect on stereoacuity recovery [14]. Third, the psychosocial impact [15], the comfort of patch wearing [16], and the long treatment periods [17] are factors associated to a poor adherence to the treatment. Finally, recurrence is estimated in more than 20% of amblyopic patients treated with occlusion. [18]

In last years, more knowledge has been acquired about the neurophysiological mechanism of amblyopia, leading to the development of new technologies for amblyopia management, including new computerized treatments based on perceptual learning (PL) and dichoptic training (DT). Perceptual learning and dichoptic therapy are supported by neurophysiological findings which show that videogames activate the neuro-modulatory pathways and improve attentional skills [19], leading to changes in cortical thickness, volume of the gray matter, and other brain areas [20]. Perceptual learning is described by some authors as the stimulation of the visual pathway through the repetition of visual tasks using Gabor’s patches as stimuli for promoting the recovery of VA and CS [21, 22]. Portela-Camino et al. reported that perceptual learning with random-dot stimuli is also effective for improving stereoacuity in amblyopic patients [23]. Dichoptic therapy consists of visual exercises in which the subject is wearing polarized or red-green glasses, with some stimuli seen by the amblyopic eye and some others by the fellow eye, whereas the background is seen by both eyes. Then, fusion is required for seeing the whole image and performing the visual task required [24]. Portela-Camino et al. [23] reported a significant stereoacuity improvement with PL based on random-dot stimuli, and Abdal et al [25]. showed that dichoptic therapy using specific software could be useful for VA and stereopsis recovery in amblyopic children.

Recently, a review of the scientific literature showed that vision therapy with perceptual learning and dichoptic therapy can add three benefits to the treatment of anisometropic amblyopia [26]. First, visual training (VT) with monocular treatments and binocular approaches have shown good efficacy for VA recovery [27, 28], even when compared with the only use of glasses or a placebo treatment [27]. VT has also shown potential benefits in adults [29, 30], with even an improvement of CS in adults after cataract surgery with multifocal IOL implantation [31]. Furthermore, Singh et al. reported better results with monocular videogames combined with patching than only using patching [27]. Likewise, in patching-resistant subjects, our research group suggested that combining perceptual learning with patching could be also an option for VA and CS recovery [32]. Second, regarding the dose–response coefficient, it is estimated that occlusion needs around 120 h for the recovery of 1 line (0.1 logMAR) of VA, while some articles suggested that only approximately 10 h of VT are required to achieve this same recovery [33]. Third, the adherence to VT seems to be better than patching. Stewart et al. [34] reported that compliance of patching is between 33 and 58%, while compliance with VT using monocular or dichoptic treatments varies from 50 to 88.6% depending on the study [23, 32, 33, 35], with a decrease of this compliance over time [32]. Therefore, VT combined with patching could be a promising option to accelerate the treatment period, optimize the results, and improve the adherence to treatment during the visual rehabilitation in amblyopia.

In strabismic amblyopia, there is not enough scientific evidence on the results of VT, although there are some authors mentioning the potential benefits of this therapeutic option in this type of amblyopia [36]. Hess and Thompson [37] suggested that active binocular treatments may be a promising treatment, Barret et al. [38] highlighted the relevance of a binocular approach for strabismic amblyopia, and Molina-Martín et al. [39] reported that occlusion with VT can be an option for strabismic amblyopia with angles of esotropia lower than 12 prism diopters.

Despite the benefits of perceptual learning and dichoptic therapy for the rehabilitation of amblyopia, there are some points that must be considered. First, the stimuli and psychophysical method used in perceptual learning therapy should be appropriate. Gabor’s patches and letter optotypes are the most used stimuli in PL experiences in amblyopia. Likewise, it is important that the stimuli are adapted adequately according to the patient’s baseline characteristics, with changes during therapy adapted to the level of visual improvement experienced in the amblyopic eye [40]. Second, adherence to the treatment is crucial for the visual improvement of the patient. Therefore, the use of videogames could be useful to maintain the attention of the patient and avoid boredom during the treatment sessions [26, 32, 33, 40]. Third, at present, there is not enough evidence supporting the prescription of VT without patching in all cases, and therefore VT could be proposed as a complement for passive treatments, such as patching or atropine, with the aim of promoting visual rehabilitation and accelerating the recovery. [26]

The main goal of this study was to assess the possible benefits of the combined treatment of amblyopia with patching and VT with a commercially available digital platform based on perceptual learning and dichoptic therapy in children and teenagers with resistance to occlusion over the only use of patching. Therefore, changes in visual acuity, contrast sensitivity, and stereopsis will be assessed. In addition, adherence to treatment and percentage of recurrences will be investigated as well.

## Method

### Patient selection

In this quasi-experimental multicentric study, patients were recruited from the Unit of Clinical Optometry and the Department of Ophthalmology of the Ribera Virgen de la Caridad Hospital (Cartagena, Spain) and the Advanced Clinical Optometry Unit of the Department of Ophthalmology of the Vithas Medimar International Hospital (Alicante, Spain). Best spectacle correction and occlusion were prescribed in all patients. Patients who improved their VA with the combination of glasses and patching were included in the patching group, and patients with no improvement after two consecutive follow-up exams were defined as patching-resistant and included in the VT group for receiving a combined treatment of perceptual learning and dichoptic therapy software and occlusion. For the recruitment, amblyopia was defined as a loss of 2 lines of VA in the worse eye with no structural or pathological findings and associated to amblyopiogenic factors such as anisometropia, strabismus, or both. Diagnosis was done according to the severity and type of amblyopia, as described in the “Introduction” section. Inclusion criteria were:

- Subjects below 18 years old with anisometropic, strabismic or mixed unilateral amblyopia treated with optical correction for 2 to 4 months and with VA worse than 0.1 logMAR in the amblyopic eye
- No active ocular or systemic disease
- No previous ocular surgery, except for strabismus surgery

The research adhered to the principles of the Declaration of Helsinki and was approved by the ethics committee for medical research of the Health Department of Alicante (General Hospital, Alicante, Spain) and Cartagena (Hospital General Universitario Santa Lucía, Cartagena, Spain).

### Clinical protocol

All the patients underwent an exhaustive baseline examination performed by an experienced optometrist and ophthalmologist. Eye exam included uncorrected and corrected VA with Snellen E-chart, cycloplegic and non-cycloplegic manifest refraction, cover test, ocular motility assessment, Worth 4-dot test, stereopsis (TNO stereo-test), and anterior segment and fundus examination with the slit lamp. After performing Worth 4-dot test and TNO stereo-test, binocular function (BF) score was obtained following the method proposed by Webber et al. [41] that provides a value of 5 for representing suppression, a value of 4 for representing simultaneous vision, and a value from 3.3 to 1.6 for stereopsis according to the logarithm of the value of stereoacuity in seconds of arc obtained with the test.

**Fig. 1:**
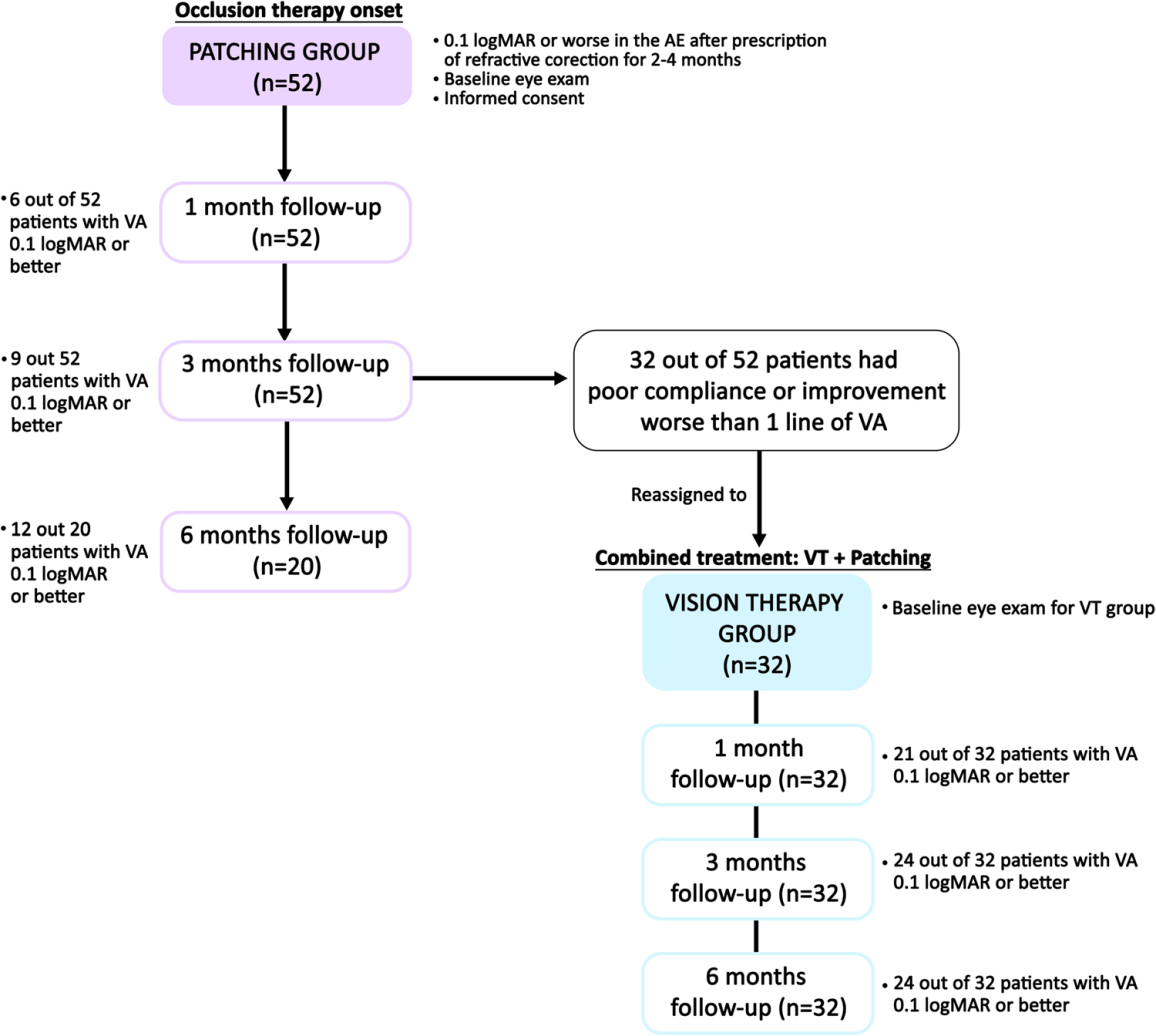
Flow chart of the clinical protocol followed in this study

Figure 1 displays the flow chart of the clinical protocol followed in this study. First, patients in the eye clinics who were diagnosed with amblyopia had the prescription of optimal spectacle correction for 2 to 4 months. In the 2- to 4-month follow-up visit, patients with VA of 0.1 logMAR or worse in the amblyopic eye were informed about the study and signed the informed consent if they agreed to participate in it. Then, those patients willing to participate in the study received patching treatment and were initially included in the patching group of the study. Patching regimen was applied following the PEDIG (Pediatric Eye Disease Investigator Group) recommendations [12, 13]. This regimen was used from the beginning of the study until patients reached 0.1 logMAR or better in their AE, regardless of the treatment group.

Then, patients were evaluated at 1, 3, and 6 months, and those who improved 1 line of VA or more in two consecutive visits and/or reached a VA of 0.1 logMAR or better in the amblyopic eye with less than 2 lines of interocular difference in VA remained in the patching group. In contrast, those patients who did not improve 1 line of VA in two consecutive visits or did not reach a VA of 0.1 logMAR or better in the amblyopic eye were considered as patching-resistant, being excluded from the patching group and included in the VT group. Also, poor compliance of patching was another inclusion criterion for the VT group. VT group received a combined therapy based on occlusion and 20 min daily of perceptual learning and dichoptic therapy. These patients were evaluated again. This eye exam carried out for patients in the VT group was considered as the baseline exam for them. Then, patients from the VT group were reviewed at 1, 3, and 6 months after initiating the treatment. Both treatments continued until the resolution of amblyopia (VA of 0.1 logMAR or better in the amblyopic eye with less than 2 lines of interocular difference in VA) or at the end of the follow-up period if no visual changes were detected in two consecutive follow-up visits. The baseline visit for the patching group was the eye exam after optical correction when occlusion was prescribed, and was compared with the baseline visit for the VT group that was performed after 3 months of patching, when patients showed resistance to occlusion and VT started. Fifty-two patients were included in the study, 32 in the VT group and 20 in the patching group.

In the VT group, changes in photopic contrast sensitivity were analyzed for 0.5, 1, 2, 4, 8, and 16 cycles/degree using the contrast sensitivity test of VisionaryTool (www.visionarytool.com), which consists of a four forced-choice answer test using a staircase psychophysical method. In strabismic patients, surgery, prism, or addition were prescribed to obtain bifoveal fixation before binocular exercises. Regular follow-up examinations of VA, refraction, and stereopsis were performed in the two hospitals involved at 1, 3, and 6 months after the beginning of the treatment. Lower values of VA and stereopsis measured with Snellen chart and TNO test, respectively, and higher scores of CS measured with the VisionaryTool indicated an improvement of amblyopia. Finally, adherence to the treatment was assessed as the percentage of the training performed versus the estimated.

### Vision therapy software: VisionarySuite

**Fig. 2:**
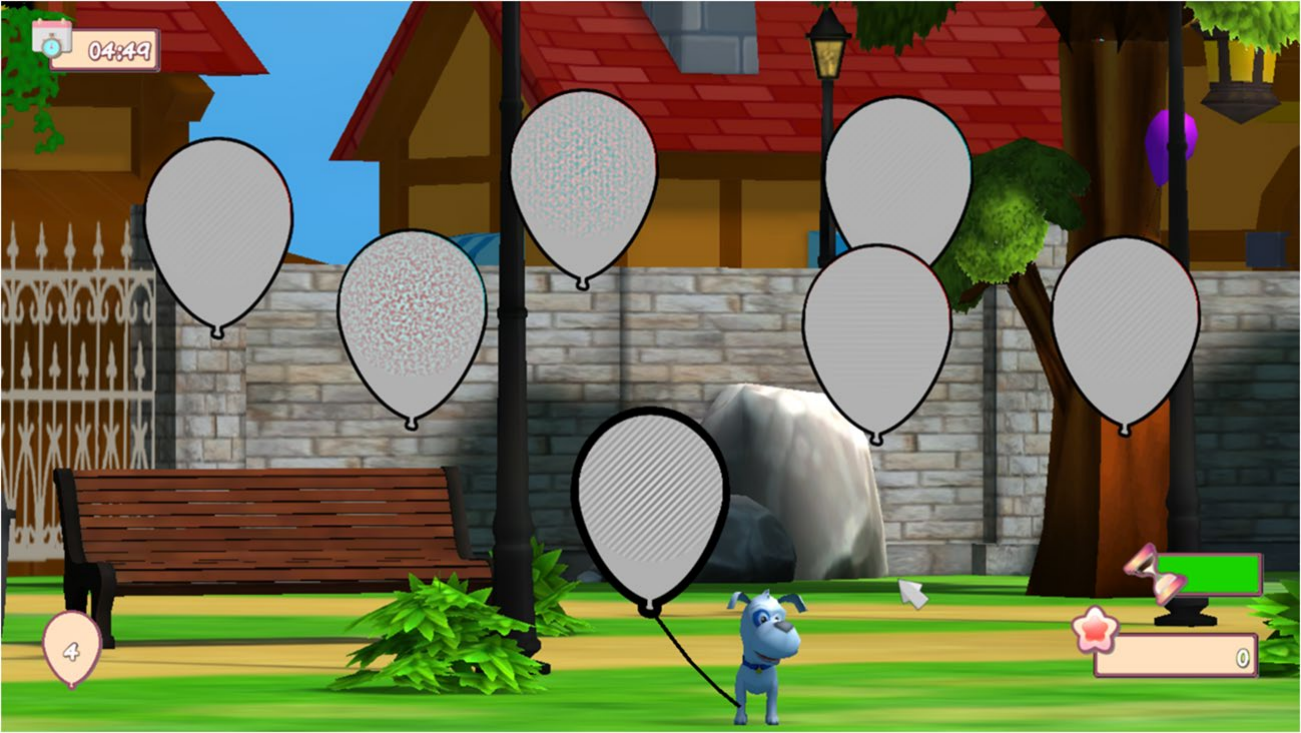
Contrast and orientation detection of Gabor’s patches with the VT system used

**Fig. 3:**
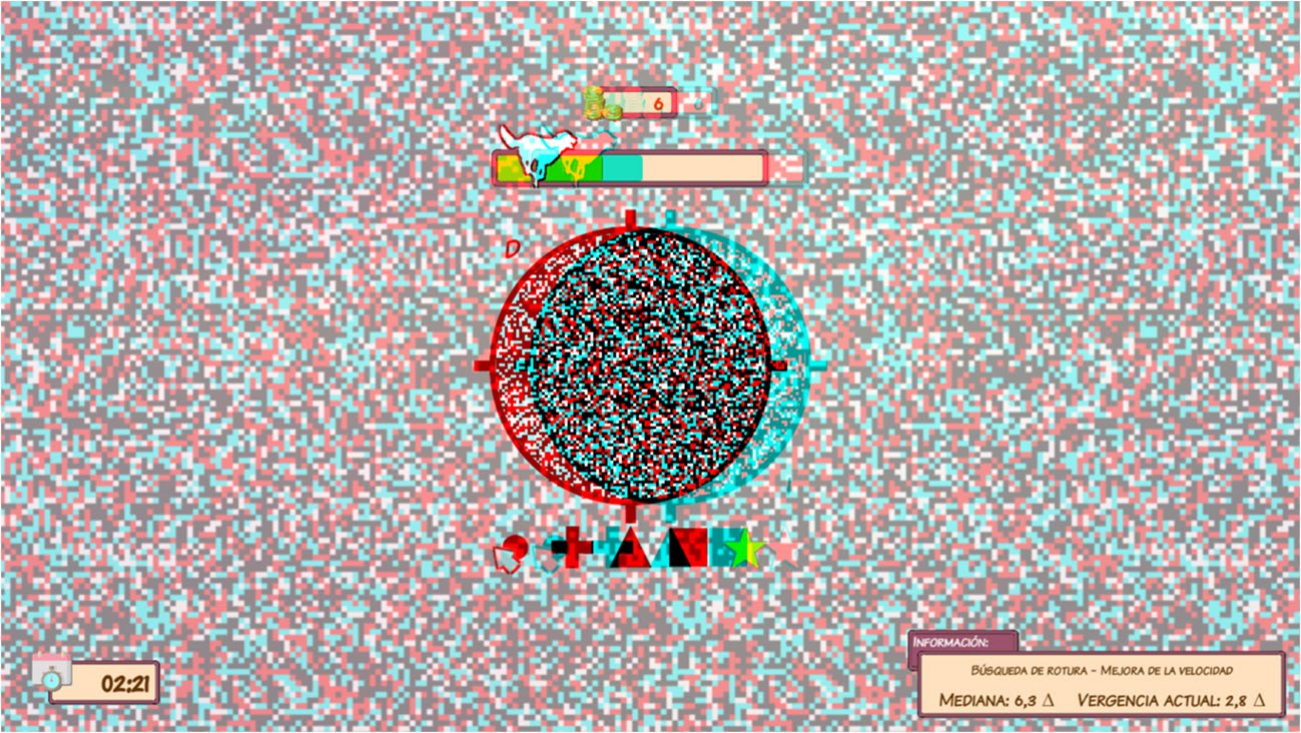
Vergence exercise with random-dot stimulus with the VT system used

**Fig. 4:**
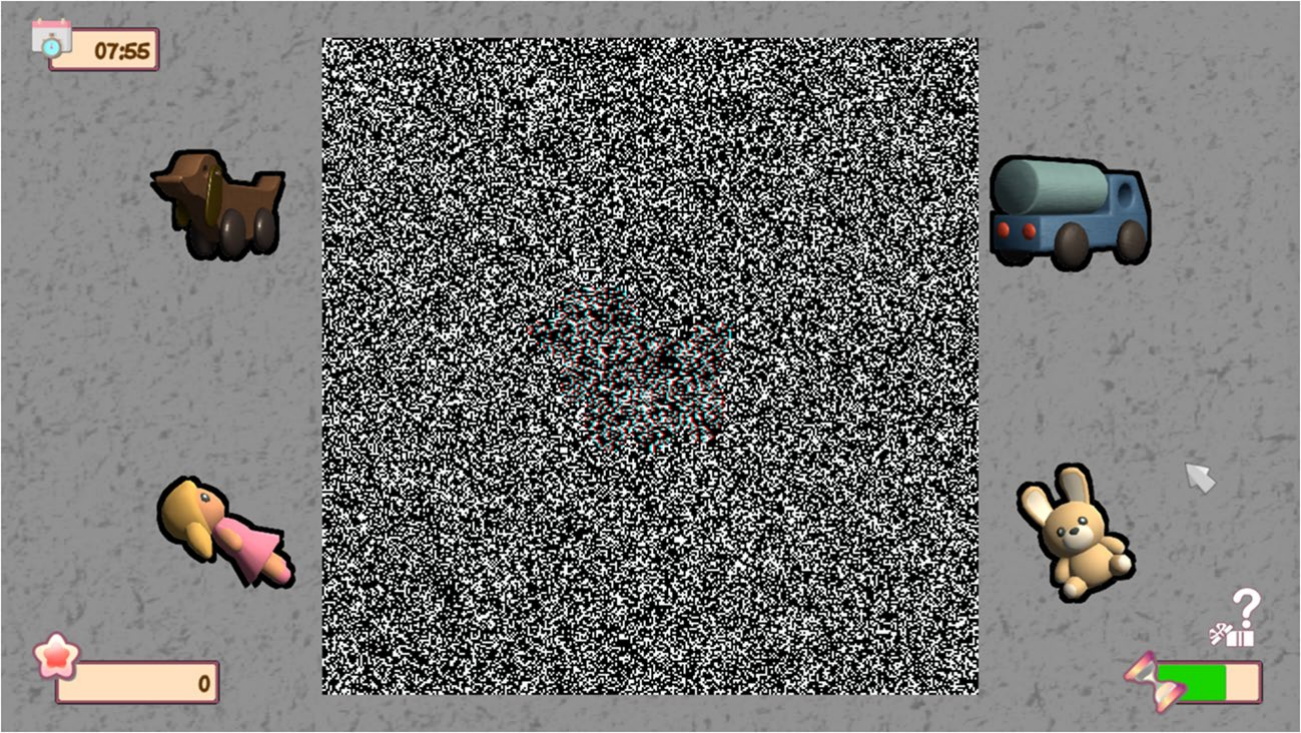
Stereopsis exercise with random-dot stimulus with the VT system used

VisionarySuite is a software from VisionaryTool based in perceptual learning and dichoptic therapy that has CE marking and has been previously used in other studies about amblyopia [23, 35, 42]. This software is downloaded and set up by the optometrist in the personal laptop of the patient. Before starting the VT procedure, data about the screen size, type of anaglyph mode, and which is the amblyopic eye was introduced for an adequate configuration and presentation of stimuli. VT exercises are subdivided into 3 main groups: group 1 for VA, CS, and fusion stimulation in which Gabor’s patches are used in different dichoptic environments (Fig. 2), group 2 including vergence exercises based in a digitalization of the conventional anaglyph exercise (Fig. 3), and group 3 including stereopsis exercises based in random-dot or contour stereopsis stimuli (Fig. 4). All exercises are performed with cyan-red glasses using the red filter in the amblyopic eye. Likewise, stimuli are adapted to patients’ answer following a staircase physiological method, with increasing spatial frequency and decreasing contrast if the patient provides correct answer during the exercise and vice versa. In the current study, VT was divided into 2 phases according to patients’ baseline parameters. In phase 1, the aim was to increase VA and CS along with the binocular fusion and therefore group 1 exercises were prescribed until reaching VA of 0.2 logMAR or better. Phase 2 was focused on improving binocular vision and stereopsis, and for such purpose group 1 exercise time was reduced to 5 min and group 2 and 3 exercises were prescribed for another 15 min to complete the whole VT session. Daily training with VisionarySuite was conducted at home and monitored remotely by the optometrist to assess treatment adherence using the data recorded by the software. VisionarySuite collects data about the date, time, and duration of the exercises.

### Data analysis

Data were analyzed using the software SPSS Statistics v. 20 (IBM, Armonk, NY, USA). For the analysis, type of amblyopia, previous treatment, and patching regimen were considered. Most of the data were not normally distributed, as verified with the Kolmogorov–Smirnov test. Therefore, non-parametric tests were used. Mann–Whitney *U* test was used for comparison of independent paired samples, Wilcoxon signed-rank test was used for comparison of dependent paired samples, Kruskal–Wallis test was used for the comparison of multiple independent samples, and Spearman’s rho was used for analyzing the level of correlation between different clinical variables. A two-tailed *p* value < 0.05 was considered as statistically significant. In addition, Wilcoxon effect size was calculated to obtain the effect size of the treatments.

A multiple regression analysis was used for obtaining a linear prediction equation of the change achievable in VA with treatment according to different baseline characteristics of the patient. Model assumptions were evaluated by analyzing residuals, the normality of non-standardized residuals (homoscedasticity), and the Cook’s distance to detect influential points or outliers. In addition, the lack of correlation between errors and multicollinearity was assessed using the Durbin–Watson test, the calculation of the collinearity tolerance, and the variance inflation factor.

Finally, to obtain the compliance of the treatment, the percentage of time of VT done versus prescribed was calculated and classified into 5 categories: 0–20% very poor, 20–40% poor, 40–60% moderate, 60–80% good, and 80–100% very good compliance.

## Results

### Subjects included in the study

Descriptive characteristics of the subjects included in each group of the current study are summarized in Table 1. VT group was significantly older (*p* < 0.001) and had more subjects who underwent previous treatment for amblyopia in other clinics before their inclusion in the study (*p* < 0.001) than patching group. Also, there were differences between groups according to the type of amblyopia. VT group included more subjects with anisometropic amblyopia (24 out of 32) than the patching group (9 out of 20). There were no significant differences between groups in other variables, such as gender (*p* = 1.000), patching regimen (*p* = 0.258), spherical equivalent (SE) of fellow eye (*p* = 0.836) or SE of amblyopic eye (*p* = 0.910), and baseline VA of amblyopic (*p* = 0.090) or fellow eye (*p* = 0.066). However, it should be noted that in the VT group there were 1 subject with anomalous correspondence and 5 subjects who did not undergo patching properly despite the professional prescription. Furthermore, in relation to strabismic patients, in the VT group, there were 6 patients with strabismus, 1 had constant exotropia that became intermittent after optical correction, 2 patients had partially accommodative esotropia with residual microtropia, 1 patient underwent botulinum toxin treatment for esotropia before being included in the study, and 2 patients had accommodative esotropia that was adequately corrected with optical correction. In the patching group, out of the 11 strabismic patients, 3 patients had non-accommodative esotropia, 2 patients had constant exotropia, 5 patients had accommodative esotropia that was corrected with optical correction, and 1 patient had partially accommodative esotropia with residual microtropia after using glasses.

**Table 1:** Descriptive characteristics of patients in VT group and patching group. *P*-values with asterisk represent those corresponding to statistical significance

	VT group (*n* = 32)	Patching group (*n* = 20)	*p* value
Age	7.88 ± 2.71	4.80 ± 1.24	< 0.001*
Gender (M/F)	16/16	10/10	1.000
Amblyopia type (aniso/strab/depriv)	24/6/2	9/11	0.019*
Patching regimen	3.68 ± 1.33	4.40 ± 1.73	0.258
Previous treatment (yes/no)	21/11	3/17	< 0.001*
FE SE	2.18 ± 2.00 (− 1.75 D, + 6.38 D)	2.55 ± 2.64 (− 0.75 D, + 8.00 D)	0.836
AE SE	2.50 ± 5.12 (− 21.50 D, + 7.50 D)	3.57 ± 2.98 (− 2.00 D, + 8.50 D)	0.910

* Means that this value represents statistical significance

*FE SE*fellow eye spherical equivalent, *AE SE* amblyopic eye spherical equivalent

### Changes in visual acuity

**Fig. 5:**
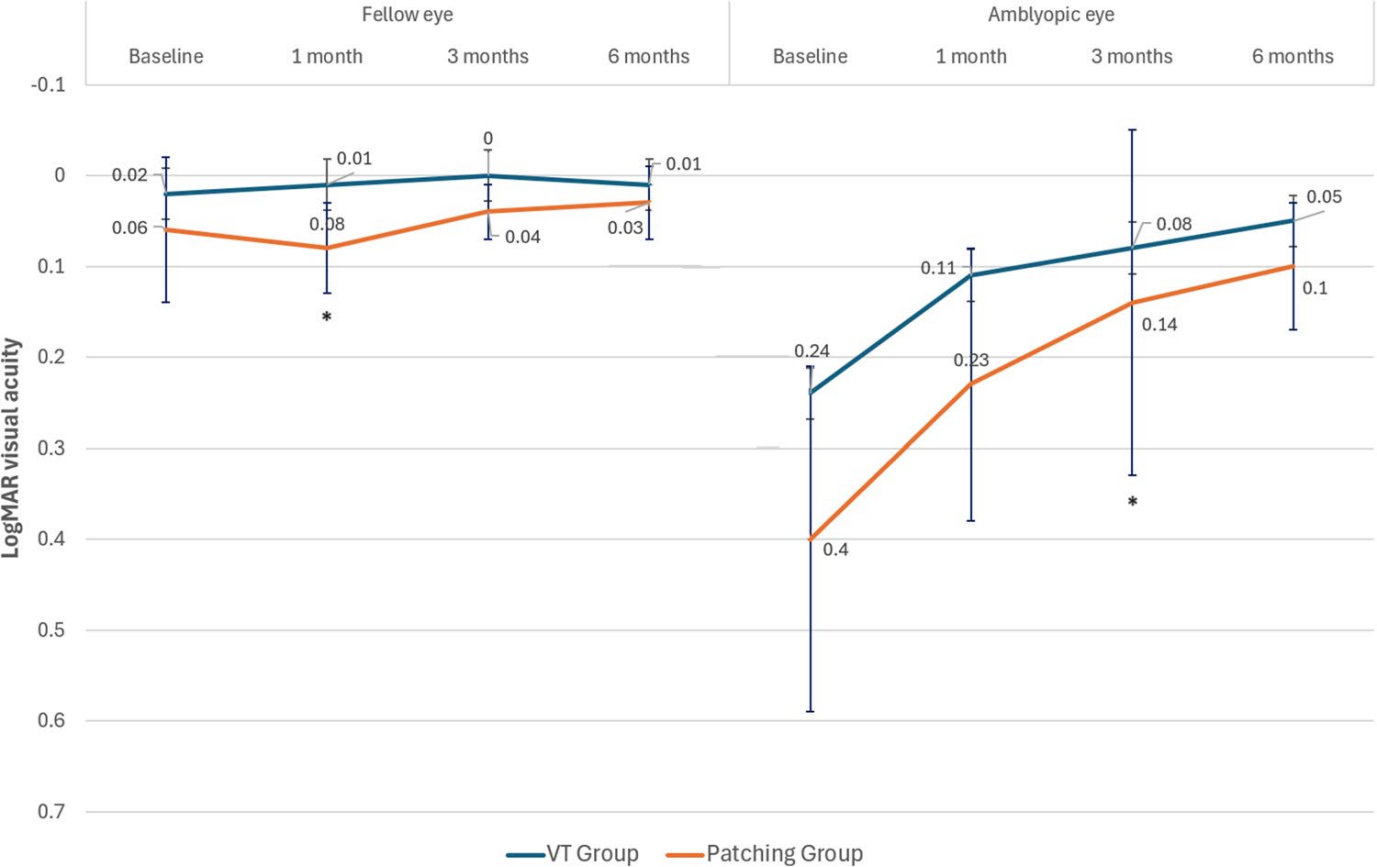
Changes in visual acuity in VT and patching groups during the follow-up in the amblyopic and fellow eye. An asterisk highlights which differences between groups reached statistical significance

**Fig. 6:**
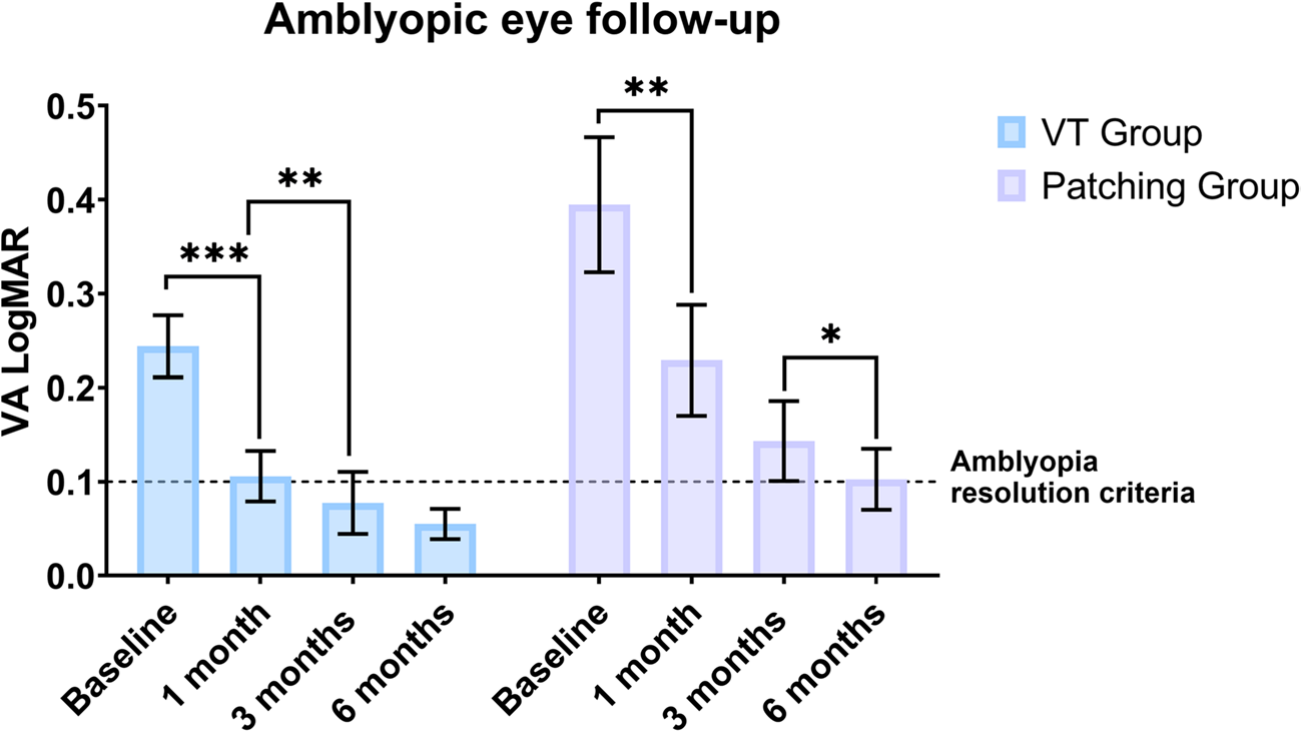
Distribution of changes in visual acuity in the amblyopic eye in VT and patching groups during the follow-up, remarking the criterion of amblyopia resolution

In Fig. 5, data on changes in VA from baseline to 6 months after initiating the treatment of both groups during treatment are displayed. In VT group, the VA of the amblyopic eye significantly improved from 0.24 ± 0.19 logMAR at baseline to 0.11 ± 0.15 (*p* < 0.001), 0.08 ± 0.19 (*p* = 0.004), and 0.05 ± 0.07 logMAR (*p* = 0.678) at 1, 3, and 6 months, respectively (Fig. 6). In patching group, the VA of the amblyopic eye significantly improved as well from 0.40 ± 0.32 logMAR at baseline to 0.23 ± 0.19 (*p* = 0.005), 0.14 ± 0.15 (*p* = 0.345), and 0.10 ± 0.13 logMAR (*p* = 0.018) at 1, 3, and 6 months, respectively. There were no statistically significant differences at baseline in the VA of the amblyopic eye between VT and patching groups (*p* = 0.090), with an improvement of this parameter with treatment in both groups, and no significant differences between them (*p* = 0.732) at the end of the follow-up. At the end of the follow-up, 36 patients (12 from the patching group and 24 from the VT group) reached a VA of 0.1 logMAR or better in the amblyopic eye.

The total improvement of VA was 0.18 ± 0.16 logMAR in the VT group and 0.31 ± 0.35 logMAR in the patching group when comparing the last visit with the baseline exam. Although patching group showed on average a higher improvement, there were no statistically significant differences between groups (*p* = 0.317). This can be due to the non-significant but lower VA of the amblyopic eye in the patching group, having these patients more range of recovery. In addition, mean VA of the amblyopic eye in VT group reached the value of 0.1 logMAR VA or better after 3 months of treatment, while in the patching group this value was achieved after 6 months of treatment.

Regarding the changes in the VA of the fellow eye, there was a slight improvement during the treatment, but not reaching statistical significance in any of the two groups. Likewise, there were no statistically significant differences between groups in this parameter, except at 1-month follow-up, but the difference was not clinically relevant.

According to the amblyopia type, there were no statistically significant differences in VA at baseline between amblyopia subgroups. However, VT group showed slightly better VA than patching group during treatment for anisometropic and strabismic cases. Specifically, the VA was significantly better in VT group after 3 months of treatment for those cases with anisometropic amblyopia (*p* = 0.007). Nevertheless, both groups reached a similar mean magnitude of anisometropic amblyopia visual recovery at 6 months (patching group: 0.04 ± 0.06 logMAR; VT group: 0.03 ± 0.06 logMAR; *p* = 0.898). In strabismic amblyopia, the VA was significantly better at 1 month (*p* = 0.041) in the VT group, although both groups improved partially and obtained similar VAs during the rest of follow-up. From a clinical perspective, the anisometropic amblyopia was rehabilitated with both treatments, but the VA recovery was faster with VT. In strabismic amblyopia, both groups showed an improvement as well, but the mean VA values after therapy did not accomplish the amblyopia resolution criteria at 6 months in any of the two groups (patching group: 0.15 ± 0.14 logMAR; VT group 0.10 ± 0.08 logMAR; *p* = 0.699).

In addition, in the VT group, there were moderate correlations between the type of amblyopia and the VA at 1 month (*r* = 0.448, *p* = 0.010), 3 months (*r* = 0.593, *p* < 0.001), and 6 months (*r* = 0.535, *p* = 0.015), and therefore patients with anisometropic amblyopia reached better VA during treatment. However, there was no correlation between amblyopia type and baseline VA (*r* = 0.145, *p* = 0.428). On the other hand, patients in the patching group showed a moderate correlation between the type of amblyopia and baseline VA (*r* = 0.519, *p* = 0.019), but without correlation with the VA achieved during and after treatment (*p* > 0.05).

Regarding the effect size of the change in VA with each type of treatment, the Wilcoxon effect size was *r* = 0.53 in the VT group and *r* = 0.44 in the patching group at 3 months. However, at the end of the follow-up, the effect size was slightly higher in VT group (*r* = 0.48) than in patching group (*r* = 0.54).

### Changes in contrast sensitivity

**Fig. 7:**
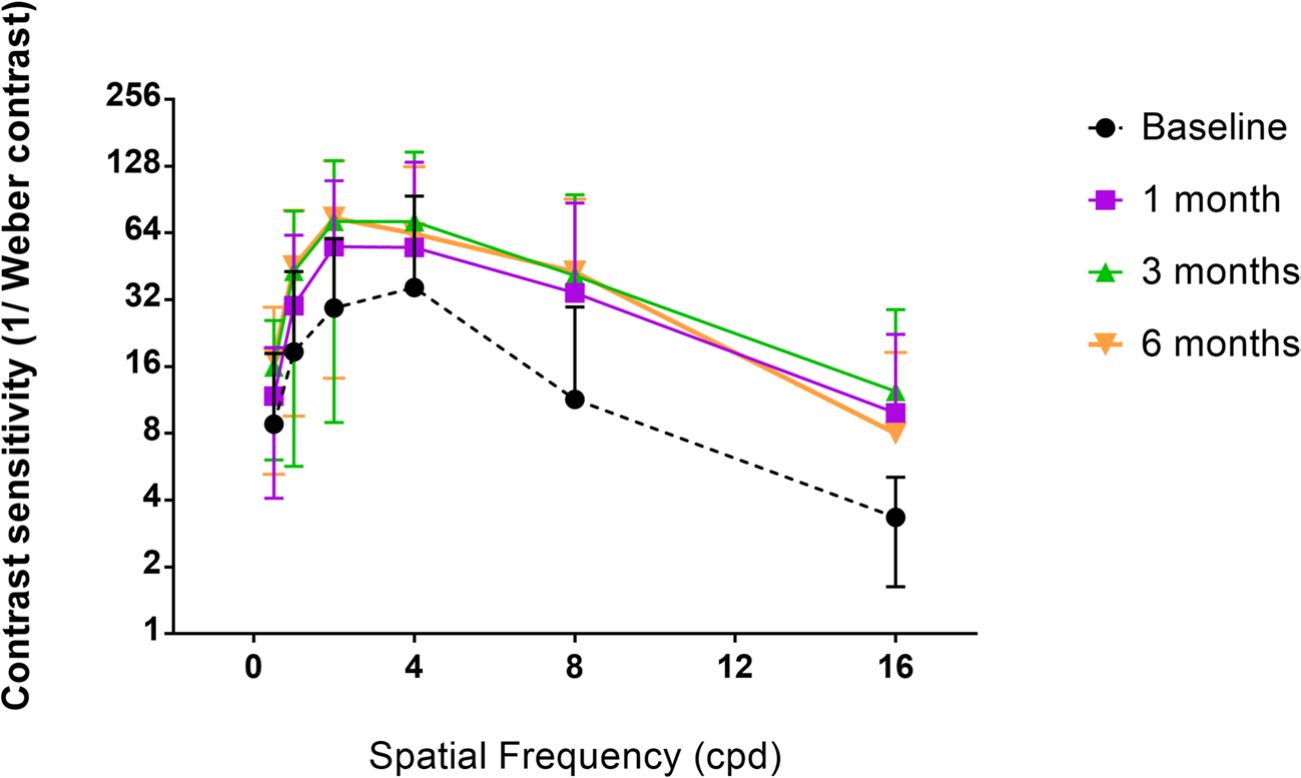
Contrast sensitivity of patients in the VT group during follow-up. Data extracted from the software VisionaryTool. The Y axis shows the contrast sensitivity based on Weber’s contrast, and the X axis shows the spatial frequencies from low (0.5 cycles/°) to high (16 cycles/°)

An enhancement in CS was observed in the amblyopic eye of the VT group (Fig. 7). This improvement was significant for all spatial frequencies at 1 month and continued improving until 3 months, although in this visit the change was only significant for 0.5 (*p* = 0.003), 1 (*p* = 0.009), 2 (*p* = 0.028), and 4 (*p* = 0.049) cycles/°. In the last visit, after 6 months, CS significantly increased as well in 0.5, 1, 2, and 8 cycles/°, but decreased in 4 (*p* = 0.037) and 16 (*p* = 0.673) cycles/°. Nonetheless, there was significant improvement in 0.5, 1, 2, 8, and 16 cycles/° from the baseline until the end of follow-up, with mean changes of 7.00 ± 5.57 for 0.5 cycles/° (*p* = 0.001), 25.94 ± 25.64 for 1 cycles/° (*p* = 0.001), 42.81 ± 39.02 for 2 cycles/° (*p* < 0.001), 33.31 ± 54.52 for 4 cycles/° (*p* = 0.004), 20.40 ± 22.50 for 8 cycles/° (*p* = 0.002), and 1.47 ± 2.85 for 16 cycles/° (*p* = 0.020), respectively.

### Binocular function score

**Fig. 8:**
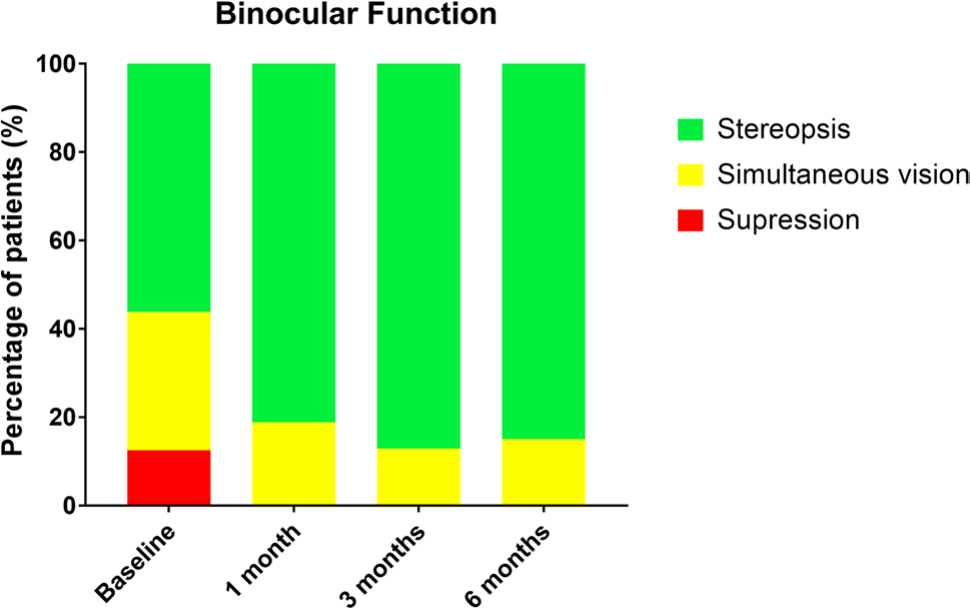
Changes in binocular function of patients in the VT group during follow-up. Percentage of patients with suppression (red), simultaneous vision (yellow), and stereopsis (green) from baseline to 6-month follow-up. After 1 month of treatment, no patients had suppression and more patients reached stereopsis

In the VT group, binocularity experienced a relevant change, improving significantly after the direct stimulation of fusion and stereopsis with dichoptic therapy (Fig. 8). Before treatment, 12.5% patients had suppression of the amblyopic eye, 31.2% subjects had simultaneous vision, and 56.2% subjects showed stereoacuity between 40 and 2000 arc sec. After 1 month of treatment, there was a significant improvement (*p* = 0.002), and as consequence, 81.2% subjects presented stereoacuity, 18.7% subjects had simultaneous vision, and any subject had suppression. At 3 and 6 months of follow-up, results were stable and there were no significant changes in BF.

### Compliance and duration of the treatment with VT

The treatment varied among subjects, performing all subjects at least 3 months of VT, with 75% of patients reaching a VA of 0.1 logMAR or better in this period. The mean time of VT at home performed was 7.7 ± 2.8 h in the first month, 7.0 ± 3.1 h in the second month, and 5.8 ± 3.5 h in the third month. Consequently, comparing the VT prescribed with the VT performed at home, compliance was 72.4% in the first month, 68.9% in the second month, and 56.8% in the third month. Compliance was poorly correlated with the VA at 3 months in the VT group (*r* = 0.383, *p* = 0.036).

### Correlations

VA showed no significant correlation with CS in VT group (*p* > 0.05). In VT group, baseline VA was strongly correlated with the total VA improvement (*r* =  − 0.770, *p* < 0.001), and VA at 1 month (*r* = 0.625, *p* < 0.001), poorly correlated with VA at 3 months (*r* = 0.386, *p* = 0.032) and no correlated with VA at 6 months (*r* = 0.107, *p* = 0.654). In the patching group, baseline VA was strongly correlated with the total VA improvement (*r* =  − 0.847, *p* < 0.001), but no with the VA reached during follow-up.

Baseline BF was significantly correlated with BF (*r* = 0.567, *p* = 0.001) and VA (*r* = 0.469, *p* = 0.007) at 1 month, BF (*r* = 0.586, *p* = 0.001) and VA (*p* = 0.574, *p* = 001) at 3 months, and BF at 6 months (*r* = 0.719, *p* < 0.001). Besides this, BF was also correlated with amblyopia type at baseline (*r* = 0.552, *p* = 0.001) and at 6 months (*r* = 0.563, *p* = 0.010). Age and gender were not found to be correlated with VA improvement in VT group. However, in patching group, there was a significant correlation between age and total VA improvement (*r* = 0.542, *p* = 0.037). In addition, previous treatment was correlated with total VA improvement in VT group (*r* = 0.512, *p* = 0.021), but not in the patching group (*p* > 0.05).

### Multiple linear regression analysis

Multiple linear regression analysis confirmed that a prediction equation of the visual improvement achievable at 1 month with the combined treatment of VT and patching could be obtained (*p* < 0.001, *R*^2^ = 0.747, Durbin–Watson: 2.153):

ΔVA = 0.100 − 0.060*(Amblyopia Type: 1, anisometropic; 2, strabismic) + 0.732*(Baseline VA) − 0.28*(interocular difference VA).

The normality of the unstandardized residuals distribution (*p* = 0.131) and the absence of outliers confirmed the homoscedasticity of this model. Similarly, no multicollinearity was detected in the model (VIF between 1.114 and 1.560).

Similarly, a prediction equation of the visual improvement achievable at 3 months with the combined treatment was obtained (*p* < 0.001, *R*^2^ = 0.850, Durbin–Watson: 2.620):

At 3 months: ΔVA = 0.161 − 0.093*(Amblyopia Type: 1, anisometropic; 2, strabismic) + 0.925*(Baseline VA) − 0.045*(interocular difference VA).

The normality of the unstandardized residuals distribution (*p* = 0.200) and the absence of outliers confirmed the homoscedasticity of this model. Similarly, no multicollinearity was detected in the model (VIF between 1.105 and 1.589).

## Discussion

Our results showed that a combination of VT and patching (VT group) improved VA in a group of patients where 21 out of 32 were patching-resistant and significantly older than patients in the patching group. In addition, VT group maintained the results during the 6-month follow-up. Liu et al. [43, 44] and Hernández-Rodríguez et al. [32] obtained similar results comparing between children with or without patching history, and Singh et al. [27] showed that combined treatment with a monocular videogame can achieve better VA than only patching. Besides, many authors also suggested that a combination of passive and active treatment could be useful, although there is still a lack of robust evidence [36, 45, 46]. Furthermore, in case of using VT, it is crucial the selection of the appropriate stimuli and psychophysical adaptive method to stimulate the visual function close to each patient’s threshold, as suggested in the scientific literature. In this study, the software used stimulates the amblyopic eye using Gabor’s patches in a dichoptic environment and random-dot stimuli, with an adaptation of the level of difficulty of visual tasks to the patient’s answer, following the recommendations from the peer-reviewed literature. [47]

In our sample, anisometropic amblyopes showed in both groups a mean visual recovery consistent with the criterion of amblyopia resolution defined, although in the VT group, this criterion was reached at 3 months, while in the patching group it was achieved at 6 months. According to this, VA seems to improve faster in the VT group. This shortening of the treatment period was previously suggested by Gambacorta et al. [33] who reported that an improvement of 0.2 logMAR in VA could be achieved faster using VT compared to patching. Concerning patients with strabismic amblyopia, they experienced a similar VA improvement in both groups, with no significant differences between them and mean values of visual recovery not accomplishing the criteria of amblyopia resolution. Molina-Martín et al. [39] reported similar results, but with slightly better VA in patients who underwent combined treatment with VT, patching and prisms or surgery. Other authors have also observed a worse response in VA to PL in strabismic amblyopia than in anisometropic amblyopia [33, 48, 49]. This could be due to the different neural mechanisms altered in strabismus, such as the greater binocular impairment, sensory correspondence, or eccentric fixation.

The effect size was better in the VT group after 3 months, but slightly worse at the end of follow-up. Nevertheless, the combination of patching and VT in a group of patching-resistant patients had a similar effect size than the only use of patching in children with a good response to occlusion, which is consistent with the results of a previous retrospective study. [32]

CS improved in the VT group during treatment, although this improvement was greater within the first month of treatment, with only slight improvements afterwards. This course of CS changes after VT is consistent with that reported in previous studies using perceptual learning-based software [32] and even software combining perceptual learning and virtual or augmented reality [50]. It must be mentioned that other authors did not find significant changes in CS with VT in amblyopia, which can be attributable to several factors such as limitations of their studies, including the size of the sample [27, 51]. At the end of the follow-up, a small in magnitude but statistically significant reduction was found in CS in VT group for the spatial frequencies of 4 and 16 cycles/°. This may be related to some level of regression of the visual function improvement achieved initially with the visual training program not affecting the level of visual acuity but having an impact on visual quality. This should be confirmed in future long-term studies. Indeed, more studies are needed to confirm the long-term stability (more than 12 months of follow-up) of the outcomes obtained with the combination of PL and DT in amblyopia.

From a neurophysiological perspective, morphological and functional changes in primary visual cortex in amblyopia are related to some visual deficits, such as reduced VA, decreased CS, and crowding. Reduced VA is associated with a reduction of the V1 activation and the enlargement of receptive visual fields. VA is also related to crowding which takes place in V1 and seems to be associated with cortical neuron insufficiency, abnormal lateral interactions, and elevated cortical noise [47]. The discrimination of Gabor’s patches is a selective mode of stimulating orientation and contrast perception, which are visual functions processed primarily in V1 [47]. Some authors suggested that a better performance of orientation and contrast recognition during visual tasks might be transferred to an improvement in VA and CS, being also correlated with positive functional magnetic resonance imaging and diffusion tensor imaging changes [52, 53] and steady-state visual evoked potentials. [54]

Besides changes in monocular function, BF also improved in our study. All patients reached some level of binocularity and most of them improved their baseline stereopsis after VT, as has been also reported by Liu et al. [45], Portela-Camino et al. [23], Martín-González et al. [35], and Molina-Martín et al. [39], and has been also suggested in a previous review by Hou and Nicholas [55]. Changes in binocularity should be one of the main goals in amblyopia treatment since amblyopia should be understood as a binocular anomaly with unilateral and bilateral deficits. Hence, interocular suppression due to the attenuation of the amblyopic eye signal and its inhibition by the fellow eye play an important role in the recovery of amblyopic patients [56]. For this reason, binocular therapies are a promising treatment option [26], and the use of a dichoptic environment during VT sessions in amblyopia can have a positive impact on BF [25, 45]. Additionally, rebalancing the visual system and improving the binocular summation by stimulating binocular fusion and stereopsis through dichoptic training and random-dot stimuli lead to an activation of early visual cortex and consequently to an improvement of BF.

During the follow-up, compliance was good during the first 2 months (72.4% and 68.9% in the first and second months, respectively), but it became moderate during the third month (56.8%), possibly due to the boredom of performing repeated tasks with the videogames, leading to a loss of motivation. Gambacorta et al. [33] reported a compliance > 60% when VT was prescribed. Adherence to the treatment is associated with the improvement of VA achieved with it, being better with VT compared to patching (compliance below 60%) [17, 57]. Future efforts in research on amblyopia treatments should be focused on optimizing and shortening treatments, since adherence to treatments is higher in the initial phases of the VT program.

Besides all the findings discussed, in the VT group of the current series, baseline VA and VA improvement were correlated, indicating that more range of visual recovery could be obtained in those eyes with worse baseline VA. This seems coherent considering that there was more room for improvement in those eyes with more visual limitation. Despite this, baseline VA was positively correlated in this group with VA at 1 month and VA at 3 months, indicating that although more visual recovery can be achieved in severe amblyopia, the value of post-therapy VA is still more limited in these cases during the first phase of the treatment, but not at its end. In the patching group, a strong correlation between baseline VA and total improvement was also obtained. Concerning BF, the baseline value was positively correlated with BF and VA at 1, 3, and 6 months, as well as with amblyopia type and VA at 1 and 3 months. Therefore, better binocularity at baseline was associated to better BF and VA during the follow-up, although with worse values in strabismic amblyopia compared to anisometropic cases. Age was not correlated with the total improvement of VA in the VT group, but it was moderately and directly correlated in the patching group. Likewise, older children seemed to have greater improvement in this sample of patients. This finding is not coherent with the knowledge about neuroplasticity, age, and amblyopia recovery, but this may be due to the level of compliance, which could be better in older children playing with the software used for VT.

From our knowledge, this is the first study proposing a predictive model for improving visual function in amblyopia using a combined treatment of VT and occlusion in patch-resistant patients. This kind of models gives information about the average duration of VT, or the prognosis of visual function improvement expected during treatment. The improvement in VA is not equal throughout the entire process, since the increase in VA was greater at the beginning. The linear prediction equation of the VA improvement according to the baseline characteristics (amblyopia type, baseline VA, and interocular difference in VA) was obtained at 1 month, but not at 3 months. Based on this, we hypothesized that VT appears to have a period of effectiveness after which VA seems to be stable. Therefore, the linear model does not fit after the first month. In any case, the linear predicting model should be validated and refined in future studies including larger samples from several clinical centers.

There is a great heterogeneity in the protocols of VT in scientific literature. Some authors propose sessions of 1 h or more of perceptual learning, once or more times a week [33, 54, 58], while others suggest daily sessions of approximately 20–40 min, 5 or more times per week [39, 49, 52]. In all cases, PL leads to improved function after a month of treatment, or when around 10 h of treatment have been completed.

In this study, we have proposed a combined treatment protocol of patching and 20 min per day of VT based on PL and DT (≈8 h per month). This approach addresses amblyopia through two pathways: monocular stimulation of VA and SC of the amblyopic eye, and training of fusion and stereopsis. With this procedure, we have achieved a significant improvement in VA, CS, and stereopsis in patch-resistant patients included in the study after 1 month of treatment, and resolution of amblyopia in 24 out of 32 patients at 3 months, with no recurrences at the end of follow-up. Furthermore, some studies on PL use unappealing systems for young patients, requiring weekly sessions of 1 h or more in-office. From a clinical perspective, gamification of PL and DT through serious videogames, and the possibility to conduct the treatment in daily sessions of 20–30 min at home, could be a good option for enhancing treatment adherence.

Limitations of this study are the lack of measurements of CS and stereopsis in the patching group not allowing us to perform an appropriate comparison between treatments according to the whole visual function, the period of follow-up that limits the analysis of recurrences to a period of 6 months of follow-up, and the size of the sample. Also, this is a non-randomized study of intervention, and investigators and participants were not blinded. Therefore, this should be considered as an additional factor leading to some level of bias in the results. Patient engagement with therapy may be related to many factors, such as the fun level associated to the game, if it is intuitive, if it is adapted to the preferences of the children which are age-related, the level of interaction required by the game, or even the children’s emotional status [40]. All these factors could not be controlled in this study and it may be considered as an additional limitation, but it should be considered that some of these factors are difficult to control or there are no validated questionnaires to investigate them with accuracy.

In conclusion, VT combined with patching is effective for improving VA, CS, and BF in children with amblyopia, especially in anisometropic amblyopia. This combination could be considered as an alternative for patching-resistant patients and for patients rejecting occlusion or with poor compliance. Likewise, a linear model is proposed to predict the VA improvement within the first month of treatment considering the type of amblyopia and the baseline VA and interocular difference in VA. However, due to the limitations of the study, these results must be confirmed in future randomized clinical trials with longer term follow-ups and larger samples, allowing the differentiation according to the type of amblyopia and the assessment of not only VA, but also stereopsis, CS, and recurrences.
